# SOX15 and other SOX family members are important mediators of tumorigenesis in multiple cancer types

**DOI:** 10.18632/oncoscience.46

**Published:** 2014-06-02

**Authors:** Kelsie L. Thu, Daiana D. Becker-Santos, Nikolina Radulovich, Larissa A. Pikor, Wan L. Lam, Ming-Sound Tsao

**Affiliations:** ^1^ BC Cancer Research Centre, Vancouver, B.C., Canada; ^2^ Ontario Cancer Institute, Princess Margaret Hospital, University Health Network at the University of Toronto

**Keywords:** SOX, SOX15, oncogene, tumor suppressor, development, cancer

## Abstract

*SOX* genes are transcription factors with important roles in embryonic development and carcinogenesis. The *SOX* family of 20 genes is responsible for regulating lineage and tissue specific gene expression patterns, controlling numerous developmental processes including cell differentiation, sex determination, and organogenesis. As is the case with many genes involved in regulating development, *SOX* genes are frequently deregulated in cancer. In this perspective we provide a brief overview of how SOX proteins can promote or suppress cancer growth. We also present a pan-cancer analysis of aberrant *SOX* gene expression and highlight potential molecular mechanisms responsible for their disruption in cancer. Our analyses indicate the prominence of *SOX* deregulation in different cancer types and reveal potential roles for *SOX* genes not previously described in cancer. Finally, we summarize our recent identification of *SOX15* as a candidate tumor suppressor in pancreatic cancer and propose several research avenues to pursue to further delineate the emerging role of *SOX15* in development and carcinogenesis.

## INTRODUCTION

*SOX* genes (SRY-related high mobility group (HMG) box) encode a family of transcription factors containing the DNA binding domain of SRY, the first *SOX* gene identified [1-3]. The twenty different SOX proteins identified in mammals to date can be subdivided into 8 groups (A, B1, B2, C, D, E, F, G, H) based on similarities in HMG box domains, gene structure, and the presence of specific functional domains including coiled-coil, transactivation and transrepression domains [2, 4]. Depending on the domains present and their specific binding partners, SOX proteins can either activate or repress the expression of target genes in a tissue-specific manner [1, 2, 5, 6]. Through their lineage-specific modulation of gene expression, SOX proteins are involved in embryonic development, regulating processes such as cell differentiation, maintenance of stemness, sex determination, and development of the central nervous, haematopoietic and other organ systems [1, 2, 5]. As *SOX* members are critical regulators of cellular programming, it is not surprising that disruption of these genes has been implicated in several human diseases including cancer [1, 2, 7].

### Roles of SOX proteins in cancer and cancer-associated pathways

Embryonic development is a tightly regulated process involving differentiation of cells into specialized cell types and rapid cell growth. It is well established that numerous genes and pathways with essential roles in development are frequently disrupted to promote carcinogenesis, which itself is characterized by aberrant cell proliferation and/or differentiation. The disruption of SOX proteins in various malignancies is a case in point.

### Oncogenic and suppressive roles of SOX proteins in tumorigenesis

*SOX* family members may act as oncogenes, tumor suppressor genes, or both depending on the cellular context, and can be activated or inactivated through a variety of genetic and epigenetic mechanisms including DNA copy number alterations, DNA methylation changes and aberrant miRNA expression [1, 2, 8, 9]. For example, in squamous esophageal, non-small cell and small cell lung cancers, *SOX2* acts as an oncogene and is activated through DNA amplification [10, 11]. SOX2 promotes cell proliferation, anchorage independent growth and is capable of transforming transbronchial epithelial cells [10, 11]. *SOX9* is another example of an oncogenic SOX protein that is overexpressed in multiple cancer types including colorectal, glioma, and pancreatic cancers [12-14]. In contrast, *SOX7* is downregulated via DNA deletions and methylation silencing, acting as a tumor suppressor gene in prostate, colon, lung, and breast cancers through its involvement in cell death, movement, invasion and proliferation [15, 16]. *SOX4* appears to have a context-dependent role in cancer as it is upregulated and promotes growth of leukemia, colorectal, lung and breast cancers, but is underexpressed and suppresses growth of bladder and liver cancers [9]. *SOX4* is disrupted through DNA copy gains, epigenetic changes involving DNA methylation and miRNAs, and sequence mutations [8, 9]. It mediates its oncogenic function via several mechanisms including suppression of apoptosis, promotion of metastasis, and maintenance of cancer-initiating cells. Similar to *SOX4*, *SOX2* and *SOX9* have also been shown to have tumor suppressive effects in specific cancer types (gastric cancer and melanoma, respectively), further emphasizing the context specific nature of *SOX* involvement in carcinogenesis [17, 18]. The differing actions of SOX proteins in cancer cells of various origins and genetic backgrounds likely underlie the disparate behaviors of SOX proteins in promoting or inhibiting tumor growth [9].

### *SOX* gene disruption in various cancer types

**Figure 1:**
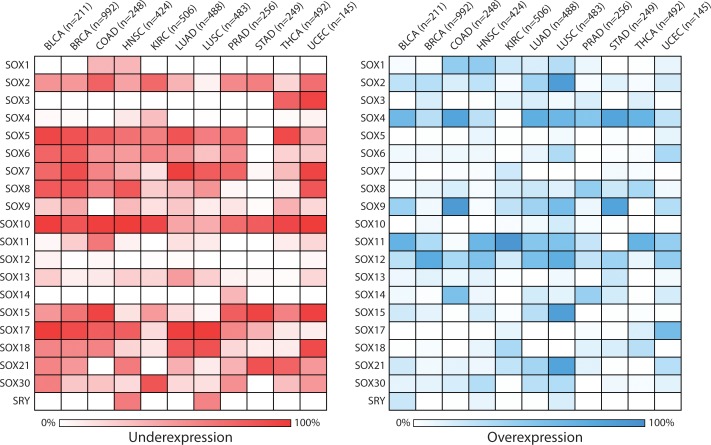
Pan-cancer analysis of SOX gene expression levels Processed Cancer Genome Atlas (TCGA) *SOX* family gene expression data for tumor and non-malignant tissues from 11 different cancer types was downloaded from the UCSC Cancer Genomics Browser (https://genome-cancer.ucsc.edu/) [92]. The number of tumor samples for each cancer type is indicated in brackets. Gene expression was classified as over- or underexpressed in individual tumors if tumor expression was at least 2-fold more or less than the average expression of available tissue matched non-malignant samples, and the frequency of expression changes across tumors of each type was calculated. Darker coloration indicates a higher frequency of alteration as indicated in the legends below each heatmap, with underexpression depicted on the left in red and overexpression on the right in blue. Genes were considered recurrently, aberrantly expressed within a particular cancer type if they exhibited a 20% or greater frequency in disruption. Cancer types are annotated as follows: BLCA - bladder urothelial carcinoma; BRCA - breast invasive carcinoma; COAD - colon adenocarcinoma; HNSC - head and neck squamous cell carcinoma; KIRC - kidney clear cell carcinoma; LUAD - lung adenocarcinoma; LUSC - lung squamous cell carcinoma; PRAD - prostate adenocarcinoma; STAD - stomach adenocarcinoma; THCA - thyroid carcinoma; UCEC - uterine corpus endometrioid carcinoma.

Not surprisingly given the known involvement of SOX members in cancer biology, a pan-cancer analysis of *SOX* expression using publically available RNA-sequencing data for 11 cancer types from The Cancer Genome Atlas (TCGA, http://cancergenome.nih.gov) revealed that *SOX* genes are frequently deregulated in several human malignancies (Figure 1, Supplemental Table 1). These results corroborate numerous reports of aberrant *SOX* gene disruption in various cancer types such as overexpression of *SOX2* in lung squamous cell carcinoma [8], low expression of *SOX10* in epithelial-derived carcinomas [19], as well as overexpression of *SOX4* and *SOX11* and underexpression of *SOX7* and *SOX17* in a variety of cancer types [1, 2, 9, 10, 16-18, 20-54]. Our analysis also identified deregulated *SOX* genes such as *SOX12* and *SOX30*, which have not been well characterized in the context of cancer, suggesting these genes may be worthwhile candidates for further investigation. Many of these RNA expression changes have also been demonstrated at the protein level as evident in the Human Protein Atlas (e.g. SOX4, SOX7, SOX10, SOX17) [55].

The most recurrently deregulated *SOX* genes, arbitrarily defined here as having a minimum 20% frequency of deregulation in at least 9 of the 11 different cancer types, included underexpression of *SOX2* (10/11), *SOX5* (10/11), *SOX6* (10/11), *SOX7* (9/11), and *SOX10* (11/11) and overexpression of *SOX4* (10/11), *SOX11* (9/11), and *SOX12* (9/11) (Figure 1, Supplemental Table 1). Interestingly, although it has been suggested that *SOX* genes are predominantly oncogenic in cancer and we found that many were overexpressed [8], we also observed recurrent underexpression of *SOX* genes in tumors relative to matched non-malignant tissues. In the 11 TCGA cancer types we considered, 13/20 *SOX* genes showed transcriptional downregulation (≥20%) whereas only 5/20 showed recurrent upregulation (≥20%) in at least 5 cancer types. Of the 13 recurrently underexpressed *SOX* genes, *SOX3*, *SOX5*, *SOX7* and *SOX10* were exclusively underexpressed (i.e. they showed overexpression frequencies <20% in all cancer types). We speculate that the prominence of *SOX* underexpression may be due to the functional redundancy of individual *SOX* genes [5, 7], as loss of function could require inactivation of multiple *SOX* family members. For example, *SOX5* and *SOX6*, members of the *SOXD* family, were frequently underexpressed concurrently in several cancer types (Figure 1). Most *SOX* genes were either over- or underexpressed within individual cancer types, though some exceptions were evident. *SOX2* and *SOX9* both exhibited frequent over- and underexpression within the same cancer type, potentially indicating their dual roles in cancer and that they could be differentially selected for in cells with different genetic backgrounds. The *SOX* genes least often disrupted at the expression level were *SOX14*, *SOX3*, and *SRY*.

As mentioned above, several genetic and epigenetic mechanisms have been associated with aberrant *SOX* expression in cancer. A similar pan-cancer investigation of TCGA genomics data for the same tumor types revealed that *SOX* genes are recurrently disrupted through copy number and methylation changes, and infrequently by sequence mutations (Supplemental Table 1). DNA amplifications were more frequent than deletions, while DNA hypermethylation was more frequent than hypomethylation at *SOX* gene loci. It is possible that these DNA-level changes underlie the prominent SOX gene deregulation we observed, although we acknowledge that additional mechanisms likely contribute to *SOX* expression as well. The prevalence of DNA and RNA alterations affecting *SOX* genes in various tumor types is strong evidence of their importance to cancer biology.

### Malignant phenotypes and cellular pathways modulated by SOX members in cancer

As described above, SOX proteins can contribute to the malignant phenotype through their abilities to regulate numerous cancer hallmarks including cell proliferation, apoptosis, survival, invasion, migration, differentiation, stemness, senescence, and angiogenesis [1, 2, 8, 9, 56]. In the context of cancer biology, Wnt/β-catenin signaling is the most well documented cellular pathway affected by SOX proteins. This pathway plays important roles in the development of multiple organs and is aberrantly activated in several cancers, driving both cell proliferation and metastasis [57, 58]. Activation of the Wnt pathway results in liberation of β-catenin from a cytoplasmic inhibitory complex, enabling it to translocate to the nucleus and bind to TCF, recruit transcriptional co-activators and stimulate expression of Wnt target genes. Numerous reports have demonstrated that SOX proteins positively (e.g. SOX2 and SOX4 in breast and colon cancers, respectively) or negatively (e.g. SOX9, SOX7, and SOX17 in colorectal cancer) regulate Wnt-mediated transcriptional activity through a variety of mechanisms, including: binding with β-catenin to prevent TCF from interacting with β-catenin, interacting with TCFs directly to inhibit them from binding β-catenin, competitive binding with TCF proteins for DNA sites, recruitment of transcriptional repressors or activators, stabilization of TCF repression, or promotion of β-catenin degradation [59, 60]. Interestingly, some SOX members (e.g. *SOX21* and *SOX9*) have also been implicated in non-canonical Wnt signaling due to their modulation of planar cell polarity signaling (PCP), which is known to contribute to tumor progression and metastasis [61-64]. Work exploring the involvement of SOX proteins in cancer through their effects on PCP may provide additional insights into how SOX disruption promotes aggressive tumor phenotypes.

**Figure 2:**
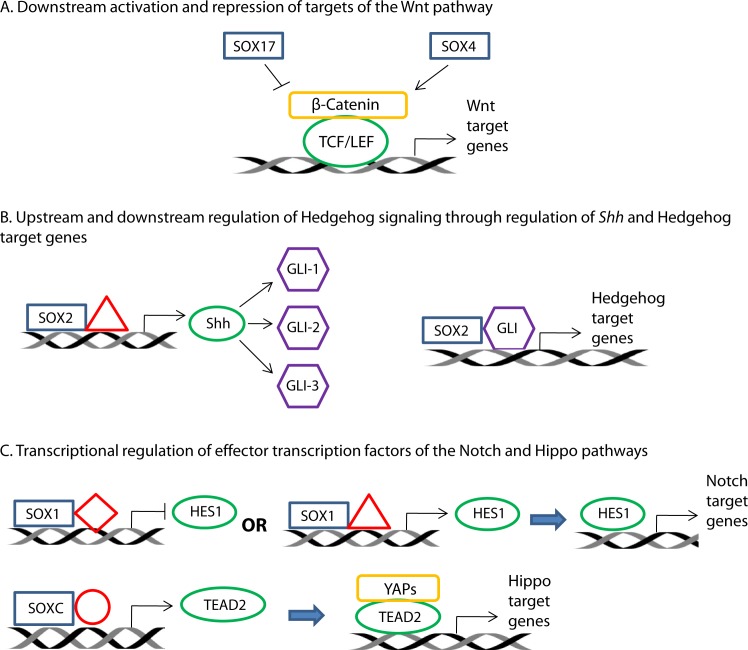
Involvement of SOX proteins in various developmental pathways that have been associated with tumorigenesis SOX members are involved in regulating several signaling pathways relevant to tumorigenesis, including the Wnt, Hedgehog, Notch, and Hippo pathways. (A) In the Wnt pathway, SOX proteins can bind β-catenin or TCF/LEF to either promote or suppress Wnt mediated transcriptional activity. (B) In the Hedgehog pathway, SOX proteins act upstream to control the expression of sonic hedgehog (Shh) and downstream through interaction with GLI to promote transcription of pathway target genes. (C) In the Notch and Hippo pathways, SOX proteins bind with other factors to control expression of their transcription factor effectors, *HES1* and *TEAD2*, respectively.

In addition to the Wnt/β-catenin pathway, *SOX* family members also have established roles in other developmental pathways including the Notch, Sonic Hedgehog, and Hippo pathways [5, 7, 65-68]. *SOX* genes can affect these pathways at both upstream (i.e. pathway stimulation) and downstream (i.e. transcriptional activity) levels, through transcriptional regulation of genes encoding pathway proteins or target genes, respectively. Figure 2 demonstrates known interactions between SOX proteins and developmental pathways implicated in carcinogenesis. SOX1 exhibits downstream regulation of the Notch pathway in neural progenitor cells by binding to the gene promoter of the *HES1* transcription factor, repressing its transcription, thereby mitigating Notch signaling and promoting neuronal differentiation [69]. In contrast, through its transcriptional activation of *HES1*, SOX1 is involved in promoting the switch from neurogenesis to gliogenesis in the ventral spinal cord [70]. In the Hedgehog pathway, SOX9 and SOX2 partner with GLI transcription factors, the downstream effectors of Hedgehog signaling to activate expression of transcription factors required for cartilage development and spinal cord neural progenitor cells, respectively [65-67]. A similar example is evident in the regulation of organogenesis via the Hippo pathway, where SOXC factors (SOX4, SOX11, and SOX12) control the expression of the transcriptional mediator of the pathway, *TEAD2* [71]. In the brain, SOX2 regulates the expression of sonic hedgehog (*SHH)*, whose corresponding protein stimulates the Hedgehog signaling cascade; this example of upstream regulation is important for stem cell maintenance in brain development [72].

### Discovery of SOX15 as a potential tumor suppressor in pancreatic cancer

We recently identified *SOX15* (also known as *SOX20*) as a potential tumor suppressor gene negatively associated with the Wnt/β-catenin pathway in pancreatic ductal adenocarcinoma (PDAC) [45]. A multi-dimensional, integrative genomic analysis of 20 PDAC cell lines revealed *SOX15* was inactivated as a result of multiple molecular mechanisms. We observed recurrent two-hit inactivation, defined as concurrent copy number loss and DNA hypermethylation associated with underexpression within an individual sample, in 45% of the cell lines assessed. Our observation of *SOX15* disruption was consistent with Knudson's two-hit hypothesis for tumor suppressor gene inactivation [73]. Following validation of *SOX15* downregulation in clinical PDAC tumors, we performed experiments demonstrating that SOX15 exerts tumor suppressor properties *in vitro* and *in vivo*. Specifically, re-expression of SOX15 in PDAC lines with undetectable endogenous levels resulted in significantly reduced cell viability and tumor growth [45]. To deduce a potential mechanism through which SOX15 may exert its tumor suppressive effects, we turned our attention to the Wnt pathway since other *SOX* family members are known to regulate Wnt/β-catenin signaling and our pathway analysis suggested a potential role for SOX15 in this pathway. Multiple different assays revealed that SOX15 expression was associated with a modest but consistent reduction in Wnt pathway activity. Taken together, our findings provide novel evidence of the involvement of yet another SOX family member in the process of carcinogenesis.

### The role of *SOX15* in developmental and cancer biology

The role of *SOX15* in cell biology and development is relatively understudied compared to other *SOX* family members, such as *SOX2*, *SOX4* and *SOX9*. Early work demonstrated *SOX15* expression in fetal brain, spinal cord, thymus, heart and adrenal tissues as well as in adult brain, lung, heart, liver, spleen, gut, small intestine, kidney, and testes tissues [74]. Knockout studies revealed that *SOX15*-null mice and embryonic stem cells are viable and grossly normal, perhaps suggesting functional redundancy with other *SOX* members [5, 7, 75, 76]. However, manipulation of *SOX15* levels in mice results in muscular abnormalities, implicating *SOX15* in the regulation of skeletal muscle development [75, 77-79]. More recent studies have suggested that *SOX15* is a potential mediator of pluripotency and stemness due to its upregulation in induced pluripotent and embryonic stem cells and mesodermal progenitor cells [80, 81].

**Figure 3:**
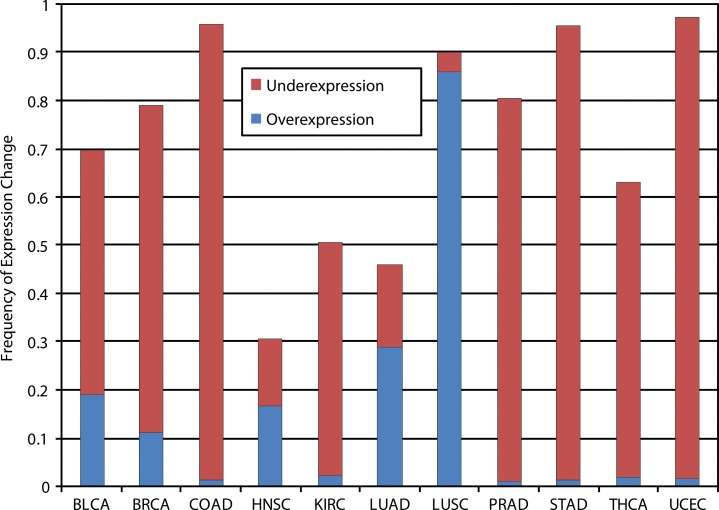
SOX15 expression status in various cancer types Frequency of SOX15 over- and underexpression in 11 cancer types, illustrating SOX15 is predominantly underexpressed in cancer with the exception of lung adenocarcinoma (LUAD) and squamous cell carcinoma (LUSC), in which it is frequently overexpressed. Cancer types are annotated as follows: BLCA - bladder urothelial carcinoma; BRCA - breast invasive carcinoma; COAD - colon adenocarcinoma; HNSC - head and neck squamous cell carcinoma; KIRC - kidney clear cell carcinoma; LUAD - lung adenocarcinoma; LUSC - lung squamous cell carcinoma; PRAD - prostate adenocarcinoma; STAD - stomach adenocarcinoma; THCA - thyroid carcinoma; UCEC - uterine corpus endometrioid carcinoma.

In cancer, *SOX15* overexpression was found to inhibit the proliferation of human testicular embryonic carcinoma cells [82]. This negative regulation of cancer cell proliferation is consistent with our results of *SOX15* expression reducing tumor growth, providing additional evidence of a potential suppressor role for *SOX15* in tumorigenesis. Further supporting this concept, our pan-cancer analysis of *SOX15* gene expression showed it was recurrently downregulated in multiple cancer types with high frequencies of disruption (≥ 80%), including colon, stomach, prostate, and uterine cancers (Figure 3). In contrast, *SOX15* appeared to be frequently overexpressed in lung adenocarcinoma and squamous cell carcinoma, potentially exemplifying the tissue-specific dependency of *SOX* gene expression and function (Figure 3). Collectively, our pan-cancer expression analysis and functional validation of SOX15 in PDAC, suggests that SOX15 may be involved in multiple cancer types.

### SOX15: Where do we go from here?

Much remains to be learned about SOX15 function in development, cell differentiation, and cancer. While we demonstrated a role for SOX15 in regulation of the Wnt/β-catenin pathway in PDAC, we acknowledge that the moderate suppression of Wnt activity does not reflect the large effect SOX15 expression had on inhibition of tumor growth, suggesting SOX15 may function through additional cellular pathways to mediate its inhibitory effect [45]. Moreover, since many *SOX* members have various roles in different cellular programs, it is possible that SOX15 is involved in normal biological processes other than just muscle development [5, 7]. We suggest three key avenues of research should be undertaken to elucidate novel roles of SOX15 in cell and developmental biology; these include identification of SOX15 transcriptional targets, understanding SOX15 expression patterns and transcriptional regulation, and identification of protein interacting partners.

Perhaps the most informative strategy for identifying novel SOX15 functions is to determine what genes it regulates (i.e. SOX15 target genes), appreciating that this may be cell-and tissue-dependent. A similar approach was recently used to infer the biological functions of SOX11 in mantle cell lymphoma and SOX2 in glioblastoma multiforme [83, 84]. SOX15 target genes could be revealed using a combination of approaches such as chromatin immunoprecipitation coupled with sequencing (ChIP-seq) to identify SOX15 DNA binding sites, and/or, genome wide expression profiling following manipulation of SOX15 levels to identify genes whose expression is strongly correlated to that of SOX15. We employed the latter approach to find cellular pathways associated with SOX15 in our PDAC study, and identified the Wnt and ERK5 signaling pathways as candidates for SOX15 regulation [45]. Although findings from genome wide approaches must be validated, they provide an excellent starting point for the identification of novel target genes.

Determining the spatial and temporal patterns of SOX15 expression throughout development will be extremely informative for identifying roles of SOX15 in developmental biology. Mapping of protein expression throughout mouse organogenesis provided insights into the role of SOX13 in multiple developmental processes, and this approach is a logical next step to further our understanding of SOX15 function [85]. Moreover, deciphering how *SOX15* is transcriptionally regulated and understanding what cellular pathways or signals activate *SOX15* gene expression could also reveal novel insights into its function. Evidence suggests that SOX gene expression can be controlled autonomously by other SOX factors, or regulated epigenetically, for example through DNA methylation or micro-RNA (miRNA) expression [5, 15, 17, 45, 80, 86, 87]; thus, knowing the biological roles of miRNAs governing *SOX15* expression could possibly shed light on SOX15 function. We also do not overlook the possibility that post-translational modifications may play an important role in mediating SOX15 behavior, as has been observed for SOX2 [5].

Lastly, it is well documented that the activity of SOX transcription factors is highly dependent on the proteins they partner with to exert their effects [88, 89]. SOX proteins may bind with completely different proteins or other SOX members. SOXB1/C/F members bind to heterologous transcription factors; for example, SOX2 partners with OCT4 (also known as POU5F1) to maintain embryonic stem cell pluripotency [90]. On the other hand, SOX5 and SOX6 can dimerize and this binding enhances their ability to bind DNA [88, 91]. The finding of SOX proteins binding downstream protein components of the Wnt pathway (e.g. TCF or β-catenin) would implicate their involvement in Wnt signaling. Thus, clues about SOX15 function could come from discerning its protein binding partners using a variety of high throughput proteomic or immunoprecipitation strategies.

## CONCLUSIONS

The reports of SOX transcription factors in the literature emphasize the critical roles *SOX* genes play in developmental and cancer biology. While some SOX proteins are well studied, we have barely scratched the surface in understanding the biological functions of many others, especially in the context of malignancy. Nevertheless, due to their regulation of stemness, pluripotency, developmental pathways, and numerous cancer processes, it is clear that *SOX* members are integral contributors to cancer biology. We have provided a snapshot of *SOX* deregulation in a variety of cancer types, revealing that *SOX* expression patterns are broadly, aberrantly expressed in cancer, heightening interest in several *SOX* members that are recurrently disrupted but have not yet been studied in carcinogenesis. We also summarized our recent finding of the relatively understudied SOX member, SOX15, as a potential tumor suppressor gene frequently inactivated in pancreatic cancer. Further work to study this gene in different cancer types and to elucidate additional mechanisms through which it may function is required to gain a better understanding of SOX15's physiological role in normal and diseased states, and could lead to the development of novel cancer therapeutic strategies [2].

## SUPPLEMENTARY TABLE


