# Supplementary material for: SOX15 and other SOX family members are important mediators of tumorigenesis in multiple cancer types
Source: Oncoscience. 2014 Jun 2;1(5):326–35. doi: 10.18632/oncoscience.46 (PMC4278306; doi:10.18632/oncoscience.46)
Supplement: Supplementary file 1 [file oncoscience-01-0326-s001.pdf]

Supplemental Table 1. Pan-cancer analysis of TCGA datasets.

Table 1A - dataset summary

Data Type	BLCA	BRCA	COAD	HNSC	KIRC	LUAD	LUSC	PRAD	STAD	THCA	UCEC
**Expression**	211	992	248	424	506	488	483	256	249	492	145
**CopyNumber**	182	985	427	388	504	493	489	197	305	494	504
**Methylation**	253*	316	166	516*	219	126	133	336*	82	500*	117
**Mutation**	99	771	NA	306	417	230	178	83	151	488	248

All processed data were downloaded from the UCSC Cancer Genomics Browser (https://genome-cancer.ucsc.edu/proj/site/hgHeatmap/)

Expression data = Illumina HiSeq, RSEM normalized counts; fold-changes were calculated in each tumour using the average expression of tissue matched non-malignant samples as a baseline; a 2-fold or greater tumour/non-malignant expression fold-change was considered aberrant expression

Copy number data = GISTIC2 processed, high-level copy number calls; log2 ratios > ±0.2 were used as a threshold for defining amplifications and deletions

Methylation data = Illumina HM27 or HM450 (indicated by * in the table above), methylation beta-values; delta-beta-values (dBV = tumour beta-value minus non-malignant beta-value) were calculated in each tumour using the average beta-value of tissue matched non-malignant samples as a baseline; dBVs > ±0.2 were considered aberrantly methylated; probes with the highest magnitude correlation with gene expression are reported

Mutation data = Somatic mutations calls; non-silent somatic mutations were defined as nonsense, missense, frame-shif indels, splice site mutations, stop codon read throughs identified in the protein coding region of a gene, or any mutation identified in a non-coding gene

Table 1B - Gene Expression

	Frequency of OVEREXPRESSION											Frequency of UNDEREXPRESSION										
**Gene**	**BLCA**	**BRCA**	**COAD**	**HNSC**	**KIRC**	**LUAD**	**LUSC**	**PRAD**	**STAD**	**THCA**	**UCEC**	**BLCA**	**BRCA**	**COAD**	**HNSC**	**KIRC**	**LUAD**	**LUSC**	**PRAD**	**STAD**	**THCA**	**UCEC**
**SOX1**	0.04	0.01	0.46	0.46	0.20	0.15	0.31	0.09	0.00	0.01	0.10	0.00	0.00	0.35	0.35	0.00	0.00	0.00	0.00	0.00	0.00	0.00
**SOX10**	0.00	0.05	0.00	0.00	0.02	0.06	0.18	0.03	0.01	0.00	0.00	0.98	0.85	0.98	0.99	0.92	0.43	0.40	0.71	0.80	0.92	0.99
**SOX11**	0.70	0.33	0.03	0.66	0.93	0.51	0.55	0.23	0.00	0.69	0.45	0.04	0.24	0.66	0.07	0.01	0.15	0.10	0.00	0.00	0.09	0.19
**SOX12**	0.28	0.76	0.36	0.56	0.19	0.58	0.73	0.25	0.40	0.09	0.46	0.07	0.01	0.04	0.00	0.01	0.02	0.01	0.00	0.01	0.00	0.04
**SOX13**	0.07	0.12	0.07	0.11	0.02	0.01	0.06	0.00	0.22	0.00	0.05	0.25	0.09	0.11	0.24	0.21	0.48	0.23	0.06	0.03	0.02	0.19
**SOX14**	0.06	0.02	0.57	0.08	0.00	0.17	0.11	0.43	0.18	0.01	0.17	0.00	0.00	0.00	0.00	0.00	0.00	0.00	0.35	0.00	0.00	0.00
**SOX15**	0.19	0.11	0.01	0.17	0.02	0.29	0.86	0.01	0.01	0.02	0.01	0.51	0.68	0.95	0.14	0.48	0.17	0.04	0.80	0.94	0.61	0.96
**SOX17**	0.00	0.01	0.00	0.00	0.10	0.00	0.00	0.03	0.01	0.15	0.61	0.99	0.91	0.80	0.79	0.18	0.98	0.99	0.58	0.37	0.11	0.09
**SOX18**	0.01	0.03	0.00	0.10	0.35	0.00	0.01	0.13	0.16	0.29	0.00	0.59	0.58	0.60	0.21	0.13	0.83	0.87	0.20	0.09	0.10	0.90
**SOX2**	0.27	0.29	0.15	0.26	0.04	0.39	0.88	0.04	0.13	0.04	0.17	0.52	0.49	0.77	0.44	0.74	0.36	0.07	0.57	0.63	0.21	0.70
**SOX21**	0.19	0.06	0.11	0.08	0.25	0.38	0.82	0.12	0.01	0.00	0.30	0.60	0.49	0.00	0.64	0.00	0.38	0.11	0.37	0.87	0.77	0.50
**SOX3**	0.00	0.16	0.00	0.01	0.10	0.16	0.06	0.16	0.00	0.14	0.01	0.00	0.00	0.00	0.00	0.00	0.00	0.00	0.00	0.00	0.77	0.95
**SOX30**	0.11	0.11	0.16	0.32	0.01	0.28	0.32	0.01	0.06	0.04	0.12	0.62	0.27	0.29	0.18	0.84	0.19	0.16	0.54	0.00	0.32	0.50
**SOX4**	0.64	0.29	0.82	0.27	0.01	0.74	0.65	0.52	0.82	0.68	0.26	0.03	0.03	0.00	0.11	0.31	0.00	0.01	0.00	0.00	0.02	0.06
**SOX5**	0.00	0.01	0.01	0.09	0.01	0.01	0.06	0.00	0.02	0.00	0.10	0.93	0.87	0.80	0.69	0.63	0.85	0.63	0.69	0.00	0.91	0.42
**SOX6**	0.06	0.07	0.07	0.07	0.01	0.10	0.33	0.02	0.02	0.03	0.35	0.76	0.81	0.54	0.49	0.60	0.51	0.30	0.55	0.00	0.23	0.26
**SOX7**	0.04	0.00	0.02	0.03	0.20	0.00	0.00	0.00	0.05	0.04	0.00	0.74	0.89	0.60	0.40	0.14	0.97	0.81	0.76	0.04	0.32	0.94
**SOX8**	0.03	0.08	0.16	0.04	0.17	0.21	0.13	0.46	0.21	0.36	0.06	0.82	0.84	0.61	0.83	0.25	0.35	0.52	0.04	0.00	0.08	0.84
**SOX9**	0.42	0.06	0.90	0.04	0.26	0.40	0.60	0.09	0.82	0.01	0.32	0.25	0.40	0.00	0.33	0.15	0.27	0.10	0.13	0.02	0.38	0.19
**SRY**	0.22	0.00	0.01	0.09	0.01	0.01	0.16	0.00	0.00	0.01	0.00	0.00	0.00	0.00	0.64	0.00	0.00	0.61	0.00	0.00	0.00	0.00

Table 1C - Copy Number

	Frequency of AMPLIFICATION											Frequency of DELETION										
**Gene**	**BLCA**	**BRCA**	**COAD**	**HNSC**	**KIRC**	**LUAD**	**LUSC**	**PRAD**	**STAD**	**THCA**	**UCEC**	**BLCA**	**BRCA**	**COAD**	**HNSC**	**KIRC**	**LUAD**	**LUSC**	**PRAD**	**STAD**	**THCA**	**UCEC**
**SOX1**	0.12	0.11	0.05	0.09	0.01	0.08	0.14	0.03	0.08	0.00	0.06	0.02	0.03	0.02	0.01	0.00	0.03	0.03	0.03	0.03	0.00	0.02
**SOX10**	0.06	0.03	0.01	0.02	0.00	0.03	0.05	0.00	0.03	0.00	0.05	0.05	0.02	0.01	0.01	0.00	0.03	0.02	0.01	0.01	0.00	0.01
**SOX11**	0.09	0.06	0.01	0.03	0.00	0.04	0.07	0.02	0.03	0.00	0.07	0.02	0.04	0.01	0.03	0.00	0.03	0.07	0.00	0.02	0.00	0.04
**SOX12**	0.04	0.03	0.02	0.03	0.00	0.04	0.07	0.00	0.04	0.00	0.03	0.03	0.07	0.06	0.03	0.00	0.05	0.05	0.02	0.01	0.00	0.03
**SOX13**	0.04	0.15	0.03	0.04	0.02	0.10	0.08	0.01	0.05	0.00	0.07	0.08	0.01	0.01	0.01	0.00	0.00	0.01	0.01	0.02	0.00	0.02
**SOX14**	0.06	0.07	0.01	0.14	0.03	0.06	0.21	0.05	0.05	0.00	0.06	0.02	0.03	0.00	0.01	0.02	0.01	0.03	0.01	0.02	0.00	0.01
**SOX15**	0.03	0.01	0.01	0.01	0.00	0.01	0.01	0.00	0.01	0.00	0.04	0.05	0.05	0.02	0.03	0.01	0.06	0.02	0.12	0.02	0.00	0.02
**SOX17**	0.08	0.10	0.05	0.06	0.01	0.08	0.08	0.02	0.05	0.00	0.07	0.05	0.05	0.04	0.02	0.00	0.04	0.05	0.04	0.04	0.00	0.01
**SOX18**	0.08	0.13	0.04	0.02	0.01	0.15	0.05	0.03	0.08	0.00	0.07	0.03	0.03	0.02	0.01	0.00	0.02	0.05	0.01	0.02	0.00	0.01
**SOX2**	0.15	0.14	0.04	0.32	0.05	0.12	0.56	0.04	0.12	0.00	0.15	0.02	0.02	0.00	0.00	0.00	0.04	0.00	0.03	0.00	0.00	0.01
**SOX21**	0.09	0.10	0.05	0.05	0.01	0.09	0.09	0.03	0.07	0.00	0.04	0.05	0.03	0.01	0.01	0.00	0.02	0.03	0.07	0.03	0.00	0.02
**SOX3**	0.07	0.06	0.03	0.04	0.01	0.09	0.08	0.01	0.06	0.00	0.02	0.01	0.03	0.01	0.01	0.01	0.02	0.03	0.01	0.02	0.00	0.02
**SOX30**	0.01	0.05	0.01	0.01	0.25	0.03	0.02	0.02	0.03	0.00	0.03	0.05	0.03	0.02	0.03	0.00	0.05	0.04	0.02	0.04	0.00	0.02
**SOX4**	0.26	0.09	0.01	0.03	0.00	0.06	0.07	0.01	0.03	0.00	0.04	0.00	0.03	0.03	0.06	0.01	0.02	0.06	0.02	0.06	0.00	0.02
**SOX5**	0.03	0.05	0.01	0.02	0.00	0.08	0.06	0.01	0.06	0.00	0.04	0.01	0.02	0.01	0.01	0.00	0.01	0.01	0.04	0.01	0.00	0.01
**SOX6**	0.02	0.03	0.01	0.01	0.01	0.03	0.03	0.01	0.03	0.00	0.01	0.08	0.05	0.00	0.04	0.00	0.04	0.07	0.01	0.01	0.00	0.04
**SOX7**	0.02	0.02	0.01	0.00	0.00	0.03	0.01	0.00	0.05	0.00	0.01	0.07	0.09	0.06	0.07	0.01	0.06	0.07	0.12	0.03	0.00	0.05
**SOX8**	0.02	0.06	0.02	0.02	0.00	0.05	0.03	0.01	0.04	0.00	0.02	0.10	0.02	0.03	0.02	0.00	0.03	0.07	0.01	0.04	0.00	0.03
**SOX9**	0.14	0.21	0.03	0.06	0.02	0.11	0.15	0.01	0.06	0.00	0.09	0.01	0.03	0.03	0.02	0.00	0.01	0.01	0.02	0.04	0.00	0.01

Table 1D - Methylation (HM27 platform)

Illumina HM27 Array		Frequency of HYPOMETHYLATION							Frequency of HYPERMETHYLATION						
**Gene**	**Probe**	**BRCA**	**COAD**	**KIRC**	**LUAD**	**LUSC**	**STAD**	**UCEC**	**BRCA**	**COAD**	**KIRC**	**LUAD**	**LUSC**	**STAD**	**UCEC**
SOX1	cg22303211	0.00	0.00	0.00	0.04	0.00	0.00	0.00	0.68	0.83	0.15	0.15	0.14	0.04	0.95
SOX10	cg19257200	0.03	0.03	0.00	0.02	0.09	0.11	0.01	0.78	0.00	0.00	0.00	0.00	0.00	0.00
SOX11	cg08432727	0.00	0.00	0.00	0.00	0.00	0.07	0.00	0.42	0.67	0.28	0.52	0.42	0.21	0.85
SOX12	cg23922081	0.00	0.00	0.00	0.00	0.00	0.00	0.00	0.00	0.00	0.00	0.00	0.00	0.00	0.00
SOX14	cg16428251	0.00	0.00	0.00	0.00	0.00	0.29	0.00	0.72	0.54	0.26	0.53	0.65	0.17	0.74
SOX15	cg01029592	0.12	0.05	0.00	0.25	0.59	0.15	0.01	0.10	0.20	0.00	0.00	0.00	0.00	0.09
SOX17	cg02919422	0.00	0.00	0.00	0.01	0.00	0.02	0.00	0.90	0.86	0.40	0.87	0.81	0.37	0.00
SOX18	cg26825412	0.06	0.00	0.00	0.05	0.05	0.01	0.01	0.23	0.31	0.10	0.14	0.12	0.04	0.46
SOX2	cg01340005	0.00	0.00	1.00	0.00	0.00	0.00	0.00	0.03	0.14	0.01	0.03	0.00	0.04	0.03
SOX21	cg19063972	0.00	0.00	0.00	0.00	0.00	0.29	0.00	0.53	0.77	0.12	0.13	0.03	0.43	0.07
SOX3	cg02847500	0.03	0.02	0.00	0.00	0.00	0.05	0.03	0.36	0.89	0.44	0.76	0.72	0.30	0.74
SOX30	cg06200339	0.20	0.10	0.00	0.08	0.26	0.12	0.08	0.00	0.00	0.00	0.00	0.00	0.00	0.00
SOX4	cg14499797	0.00	0.00	0.00	0.00	0.00	0.00	0.00	0.00	0.00	0.00	0.00	0.00	0.00	0.00
SOX5	cg14242042	0.00	0.00	0.00	0.00	0.00	0.00	0.00	0.01	0.73	0.00	0.02	0.02	0.49	0.00
SOX6	cg22586527	0.06	0.43	0.00	0.07	0.14	0.17	0.14	0.14	0.03	0.00	0.02	0.00	0.04	0.00
SOX7	cg08056146	0.00	0.00	0.00	0.00	0.00	0.60	0.00	0.32	0.60	0.02	0.39	0.05	0.23	0.01
SOX8	cg21530890	0.00	0.00	0.00	0.00	0.00	0.38	0.00	0.47	0.48	0.29	0.36	0.10	0.30	0.21
SOX9	cg06391468	0.00	0.00	0.00	0.03	0.00	0.26	0.00	0.05	0.00	0.00	0.06	0.06	0.00	0.01

Table 1E - Methylation (HM450 platform)

Illumina HM450 Array		Frequency of HYPOMETHYLATION				Frequency of HYPERMETHYLATION			
Gene	Probe	BLCA	HNSC	PRAD	THCA	BLCA	HNSC	PRAD	THCA
SOX1	cg25476766	0.11	0.00	0.00	0.00	0.09	0.59	0.25	0.06
SOX10	cg05447556	0.02	0.01	0.00	0.00	0.00	0.00	0.00	0.00
SOX11	cg06830064	0.28	0.53	0.01	0.00	0.21	0.00	0.00	0.00
SOX12	cg10625705	0.00	0.00	0.00	0.00	0.04	0.02	0.00	0.00
SOX13	cg19779057	0.12	0.17	0.00	0.03	0.16	0.00	0.10	0.00
SOX14	cg04374393	0.08	0.00	0.03	0.00	0.11	0.38	0.15	0.33
SOX15	cg07488259	0.20	0.12	0.01	0.00	0.22	0.04	0.29	0.00
SOX17	cg02222728	0.19	0.00	0.00	0.00	0.07	0.75	0.36	0.05
SOX18	cg24199599	0.10	0.00	0.00	0.00	0.13	0.16	0.69	0.05
SOX2	cg13325919	0.12	0.19	0.07	0.11	0.01	0.11	0.33	0.03
SOX21	cg16797691	0.00	0.00	0.00	0.00	0.03	0.00	0.01	0.04
SOX3	cg06902764	0.21	0.00	0.00	0.00	0.12	0.78	0.39	0.19
SOX30	cg16313910	0.08	0.21	0.03	0.03	0.00	0.00	0.00	0.00
SOX4	cg13995516	0.00	0.00	0.00	0.42	0.09	0.31	0.05	0.12
SOX5	cg16095464	0.43	0.67	0.23	0.03	0.23	0.00	0.00	0.00
SOX6	cg21992400	0.13	0.17	0.00	0.08	0.00	0.08	0.00	0.47
SOX7	cg22272840	0.34	0.04	0.01	0.09	0.22	0.05	0.66	0.38
SOX8	cg04398526	0.17	0.64	0.03	0.05	0.13	0.00	0.00	0.00
SOX9	cg03917138	0.13	0.05	0.04	0.11	0.00	0.23	0.27	0.34

Table 1F - Non-Silent Somatic Mutations

Gene	BLCA	BRCA	HNSC	KIRC	LUAD	LUSC	PRAD	STAD	THCA	UCEC
**SOX1**	0.01	0.00	0.01	0.00	0.00	0.01	0.00	0.03	0.00	0.00
**SOX10**	0.00	0.00	0.00	0.00	0.01	0.01	0.00	0.02	0.00	0.02
**SOX11**	0.00	0.00	0.00	0.00	0.01	0.01	0.00	0.03	0.00	0.01
**SOX12**	0.02	0.00	0.00	0.00	0.00	0.00	0.00	0.00	0.00	0.00
**SOX13**	0.00	0.00	0.01	0.00	0.01	0.00	0.00	0.03	0.00	0.01
**SOX14**	0.00	0.00	0.00	0.00	0.01	0.03	0.00	0.01	0.00	0.02
**SOX15**	0.00	0.00	0.00	0.00	0.00	0.00	0.00	0.01	0.00	0.01
**SOX17**	0.00	0.00	0.00	0.00	0.00	0.00	0.01	0.03	0.00	0.03
**SOX18**	0.01	0.00	0.00	0.00	0.00	0.01	0.00	0.00	0.00	0.00
**SOX2**	0.01	0.00	0.01	0.00	0.00	0.01	0.00	0.01	0.00	0.01
**SOX21**	0.00	0.00	0.00	0.00	0.00	0.00	0.00	0.01	0.00	0.01
**SOX3**	0.00	0.00	0.01	0.00	0.01	0.01	0.00	0.01	0.00	0.02
**SOX30**	0.01	0.00	0.01	0.00	0.02	0.02	0.01	0.02	0.00	0.02
**SOX4**	0.01	0.00	0.00	0.00	0.00	0.01	0.00	0.00	0.00	0.00
**SOX5**	0.06	0.00	0.02	0.01	0.05	0.03	0.00	0.07	0.00	0.03
**SOX6**	0.01	0.00	0.00	0.01	0.04	0.02	0.00	0.05	0.00	0.04
**SOX7**	0.00	0.00	0.00	0.00	0.00	0.00	0.00	0.00	0.00	0.00
**SOX8**	0.01	0.00	0.01	0.00	0.00	0.02	0.00	0.01	0.00	0.00
**SOX9**	0.00	0.00	0.01	0.01	0.01	0.01	0.00	0.03	0.00	0.02

*No mutation data available for COAD
